# Deutsche Version der Northoff Skala für subjektives Erleben bei Katatonie (NSSC-dv): Ein validiertes Messinstrument zur Erfassung des subjektiven Erlebens bei Katatonie

**DOI:** 10.1007/s00115-023-01575-4

**Published:** 2023-12-13

**Authors:** Geva A. Brandt, Stefan Fritze, Maria Krayem, Jonas Daub, Sebastian Volkmer, Jacqueline Kukovic, Andreas Meyer-Lindenberg, Georg Northoff, Katharina M. Kubera, Robert Christian Wolf, Dusan Hirjak

**Affiliations:** 1 Klinik für Psychiatrie und Psychotherapie, Zentralinstitut für Seelische Gesundheit, Medizinische Fakultät Mannheim, Universität Heidelberg, J5, 68159 Mannheim, Deutschland; 2 Mind, Brain Imaging and Neuroethics Research Unit, The Royal’s Institute of Mental Health Research, University of Ottawa, Ottawa, ON Kanada; 3 Zentrum für Psychosoziale Medizin, Klinik für Allgemeine Psychiatrie, Universität Heidelberg, Heidelberg, Deutschland

**Keywords:** Katatonie, Subjektivität, Psychomotorik, Erleben, Psychotherapie, Catatonia, Subjectivity, Psychomotor, Experience, Psychotherapy

## Abstract

PatientInnen mit Katatonie zeigen oft starke motorische, affektive und verhaltensassoziierte Symptome, hinter welchen das subjektive Erleben häufig verborgen bleibt. In dieser Arbeit soll die eigene systematisch-empirische Untersuchung zum subjektiven Erleben katatoner PatientInnen einem deutschsprachigen Publikum vorgestellt werden. Auf Basis aktueller Evidenz und der klinischen Erfahrung der AutorInnen wurde die „Northoff Skala für subjektives Erleben bei Katatonie“ (NSSC) modifiziert, erweitert und validiert. Es handelt sich um einen einfach anwendbaren Selbstauskunftsfragebogen, bestehend aus 26 Items, die das subjektive Erleben der Katatonie in seiner gesamten klinischen Vielfalt erfassen. Insgesamt wurden 46 PatientInnen mit Katatonie gemäß Internationaler Klassifikation der Krankheiten (11. Revision, ICD-11) nach ihren subjektiven Erlebnissen während der akuten Krankheitsphase gefragt. Die NSSC zeigte eine hohe interne Konsistenz (Cronbach’s Alpha = 0,91). Der NSSC-Gesamtscore war signifikant mit der Northoff Catatonia Rating Scale (NCRS; r = 0,43; *p* < 0,01), dem Gesamtscore der Positive and Negative Syndrome Scale (PANSS; *r* = 0,30; *p* < 0,05), der Brief Psychiatric Rating Scale (BPRS; *r* = 0,33; *p* < 0,05) sowie mit Trait-Ängstlichkeit (STAI; *r* = 0,64; *p* < 0,01) assoziiert, was ihre Validität unterstützt. Eine vorläufige Validierung der NSSC ergab gute psychometrische Eigenschaften. Die NSSC ist ein nützliches Instrument für den klinischen Alltag zur Beurteilung des subjektiven Erlebens von PatientInnen mit Katatonie, um passende psychotherapeutische Interventionen bereitstellen zu können.

**Zusatzmaterial online:**

Zusätzliche Informationen sind in der Online-Version dieses Artikels (10.1007/s00115-023-01575-4) enthalten.

## Abstract (en)

Patients with catatonia often show serious motor, affective and behavioral symptoms, behind which the subjective experience often remains hidden. Therefore, this study disseminates our own systematic empirical investigation of the subjective experience of catatonia patients to a German-speaking audience of clinicians and researchers. Based on current evidence and the clinical experience of the authors, the self-report questionnaire Northoff Scale for Subjective Experience in Catatonia (NSSC) was modified, extended and validated and now consists of 26 items capturing the subjective experience of catatonia in its clinical diversity. A total of 46 patients with catatonia according to the International Classification of Diseases (11th revision, ICD-11) were asked about their subjective experience during the acute phase of the disease using the NSSC. The NSSC showed high internal consistency (Cronbach’s alpha = 0.91). The NSSC total score was significantly associated with the Northoff Catatonia Rating Scale (NCRS; *r* = 0.46; *p* < 0.05), the total score of the Positive and Negative Syndrome Scale (PANSS; *r* = 0.30; *p* < 0.05), the Brief Psychiatric Rating Scale (BPRS; *r* = 0.33; *p* < 0.05), and Trait Anxiety (STAI; *r* = 0.64; *p* < 0.01), supporting its validity. Preliminary validation of the NSSC revealed good psychometric properties. The NSSC is a useful instrument for routine clinical use to assess the subjective experience of patients with catatonia in order to provide tailored psychotherapeutic interventions.

## Einführung

Katatonie ist durch verschiedene motorische, affektive und kognitiv-verhaltensassoziierte Symptome charakterisiert [1–4] und tritt bei 9–17 % der PatientInnen mit akuten psychischen Störungen auf [5–7]. Seit Januar 2022 kann Katatonie in der 11. Revision der Internationalen Klassifikation der Krankheiten (ICD-11) als eigenständige Diagnose kodiert werden. Für die Diagnose sind mindestens drei psychomotorische Auffälligkeiten notwendig (s. auch https://icd.who.int/browse11/l-m/en), die aus den Bereichen „verminderte“, „erhöhte“, oder „abnorme“ psychomotorische Aktivität stammen. Die Diagnose der Katatonie basiert auf der Verhaltensbeobachtung und körperlichen Untersuchung, aber berücksichtigt nicht das subjektive Erleben der PatientInnen. Oft fühlen sich PatientInnen mit Katatonie ängstlich, angespannt und erleben intensive und nichtkontrollierbare Emotionen oder leiden unter starker Ambivalenz. Darüber hinaus können einige PatientInnen eine Erklärung dafür liefern, warum sie nicht in der Lage sind, sich zu bewegen, und nicht auf verbale oder nonverbale Hinweise reagieren [8]. Katatone Symptome können oft im Zusammenhang mit wahnhaftem Erleben und Halluzinationen auftreten [8]. Die PatientInnen berichten häufig, dass sie den katatonen Zustand der Akinese oder Starrheit und des Mutismus annehmen mussten, oder bizarre Bewegungen ausführen mussten, um ihre wahnhaften Überzeugungen, Halluzinationen oder Ich-Störungen zu bewältigen. Anderen PatientInnen ist nicht bewusst, dass sie nicht in der Lage waren, ihre Bewegungen auszuführen oder zu beenden [9, 10]. Einige leiden unter ausgeprägter Amnesie für diese Episode [11]. Ätiologisch diskutiert wird, ob es sich insbesondere bei akinetischen katatonen Symptomen um einen evolutionären Reaktionsmechanismus auf Furchtstimuli handelt, im Sinne eines Erstarrens („Freeze“; [12]). In der Literatur finden sich ebenfalls Berichte über einen möglichen Zusammenhang zwischen Traumatisierung und dem Auftreten katatoner Symptome, im Sinne einer tonisch-immobilen pathologischen Reaktion auf traumatische Ereignisse [13–15].

Bisher wurden Untersuchungen des subjektiven Erlebens bei PatientInnen mit Katatonie nur selten durchgeführt [16]. Über die Gründe lässt sich nur spekulieren: Erstens kann davon ausgegangen werden, dass sich KlinikerInnen und ForscherInnen primär auf die Behandlung der akuten und schweren motorischen Symptome sowie der teilweise gravierenden somatischen Nebenwirkungen wie Lungenentzündung, Mangelernährung, Dekubitus und Dehydrierung fokussierten [17, 18]. Zweitens wurden affektive Symptome lange nicht als charakteristisch für Katatonie angesehen, weil die Diagnose gemäß der 10. Revision der Internationalen Klassifikation der Krankheiten (ICD-10) oder der 5. Revision des Diagnostischen und Statistischen Leitfadens psychischer Störungen (DSM‑5) gestellt wurde. In beiden Klassifikationssystemen (und der Mehrheit der klinischen Skalen zur Erfassung der katatonen Symptome) stehen eher motorische und verhaltensassoziierte Symptome wie Mutismus, Stupor und andere bizarre psychomotorische Phänomene (z. B. Posieren, Katalepsie und wächserne Biegsamkeit) im Vordergrund [19–22]. Drittens gibt es bislang nur zwei Selbsteinschätzungsinstrumente zur Beurteilung des subjektiven Erlebens bei PatientInnen mit Katatonie. Im Jahr 1996 entwickelte Georg Northoff die „Selbstbeurteilungsskala zum subjektiven Erleben katatoner Patienten“ ([11]; Northoff Scale for Subjective Experience in Catatonia, NSSC; [23]). Diese Skala enthält 14 Elemente, die mit einem kontinuumsbasierten Ansatz gemessen werden, indem das subjektive Erleben auf einer Linie dargestellt wird. Nach Northoff et al. [11] verfügen katatone PatientInnen mit ausgeprägten motorischen Symptomen nur selten über die Einsicht in ihre akinetischen Zustände. Dafür beschreiben sie ihre Erfahrungen mit affektiven und kognitiv-verhaltensassoziierten Phänomenen als intensiv und belastend [11]. Neben der Selbstbeurteilungsskala von Northoff et al. [11] wurde bisher nur ein weiteres klinisches Instrument zur Erfassung des subjektiven Erlebens bei Katatonie entwickelt. Der von Dell’Osso et al. entwickelte Fragebogen „Katatonie-Spektrum“ (CS; [24]) besteht aus 74 Items, die in 8 Domänen unterteilt sind. Der CS ist speziell auf die Beurteilung des gesamten Katatoniespektrums zugeschnitten, von manifesten bis hin zu unterschwelligen Formen der Katatonie. Darüber hinaus wurde der CS entwickelt, um die katatone Symptomatik in den verschiedenen diagnostischen Kategorien des DSM‑5 zu messen [25] und stellt somit ein begleitendes diagnostisches Instrument dar.

Um die oben erwähnte klinische und wissenschaftliche Lücke zu schließen, hatte die vorliegende Studie vier Hauptziele: *Erstens* haben wir die ursprüngliche deutsche Version der NSSC aus dem Jahr 1996 [11] von ihrem Kontinuumansatz in eine Likert-Skala umgewandelt, um die Bearbeitung und Auswertung des Fragebogens in der klinischen Praxis zu erleichtern. Darüber hinaus wurde die NSSC um weitere katatone Symptome erweitert, die in den klinischen Fremdbeurteilungsskalen erwähnt werden, wie z. B. der Northoff Catatonia Rating Scale (NCRS; [26]), der Bush Francis Catatonia Rating Scale (BFCRS; [27]) und in der ICD-11 (https://icd.who.int/browse11). Um die Skala einem breiteren Spektrum von PatientInnen und WissenschaftlerInnen bereitzustellen, wurde die NSSC kürzlich ins Englische übersetzt [23]. Die vorliegende Arbeit greift auf Methoden, Ergebnisse und Diskussionsabschnitte zurück, die kürzlich von Brandt et al. [23] in einem englischsprachigen Journal veröffentlicht wurden. Angesichts der Bedeutung dieser Publikation für klinisch Tätige und Forschende gleichermaßen, ist es unser erklärtes Ziel, diese Arbeit auch einem erweiterten, primär deutschsprachigen Leserkreis zur Verfügung zu stellen. Dieser Ansatz [28] bietet sich an, um wissenschaftliche Erkenntnisse einem deutlich größeren Publikum zur Verfügung zu stellen und diese länder- und sprachübergreifend weiter zu disseminieren und eine noch bessere Integration von Erkenntnissen und Fortschritten aus der internationalen wissenschaftlichen Gemeinschaft zu ermöglichen. Explizit möchten wir dazu beitragen, dass die NSSC auch deutschsprachigen klinisch tätigen PsychiaterInnen und PsychotherapeutInnen zur Verfügung gestellt wird. *Zweitens* zielte diese Studie darauf ab, eine vorläufige Validität des Fragebogens in einer Stichprobe von 46 PatientInnen mit Katatonie gemäß ICD-11 zu untersuchen. *Drittens* interessierten wir uns für den Zusammenhang zwischen dem subjektiven Erleben katatoner PatientInnen und Trait-bezogener Ängstlichkeit, dem Zusammenhang mit traumatisierenden Erfahrungen in der Kindheit und Jugend, da sich in der Literatur Hinweise darauf finden, dass das katatone Erleben Ausdruck überwältigend empfundener Angst sein könnte und auch als pathologische Reaktion auf traumatische Stimuli diskutiert wurde. Außerdem war unser erklärtes Ziel in dem Zusammenhang, die Emotionsregulation katatoner PatientInnen näher zu beleuchten, da eine Subgruppe von PatientInnen mit Katatonie eine Erklärung dafür geben kann, warum sie sich lange Zeit nicht bewegen und auf externe Stimuli kaum reagieren [8]. *Schließlich* versuchten wir, die drei Domänen der NCRS (z. B. motorisch, affektiv und verhaltensassoziiert) auf der subjektiven Ebene abzubilden und frühere Erkenntnisse der NCRS und der Originalversion der Selbstbeurteilungsskala von Northoff et al. [11] zu replizieren.

## Material und Methoden

### Studienpopulation

Für diese Studie wurden insgesamt 46 rechtshändige PatientInnen, welche die ICD-11-Kriterien für Katatonie in Verbindung mit einer anderen psychischen Störung (6A40) erfüllten, untersucht. Diese Studie ist Teil einer prospektiven Studie, die sich auf mikrostrukturelle Veränderungen der weißen Substanz bei Katatonie konzentriert (whiteCAT; [29]). Es handelt sich um ein ProbandInnenkollektiv, das initial bei Brandt et al. [23] berichtet und für diese Arbeit um 18 weitere ProbandInnen mit Katatonie gemäß ICD-11 erweitert wurde. Die Informationen zu den Ein- und Ausschlusskriterien, der Erweiterung der Northoff Skala für subjektives Erleben bei Katatonie (NSSC), den klinischen Untersuchungen und der statistischen Analyse sind im Supplement zu finden.

## Ergebnisse

Die NSSC beinhaltet nach der Erweiterung insgesamt 26 Items (für PDF-Version der Skala s. Supplement). Für die Kodierung waren u. a. folgende zwei Aspekte wichtig: Item #0 diente als Klassifikator, ob sich die PatientInnen an die akute katatone Phase erinnern konnten (2: „sehr gut“, 1: „teilweise“, 0: „überhaupt nicht“), Item #26 bewertete die subjektive affektive Valenz in Bezug auf den akuten katatonen Zustand. Die PatientInnen wurden gefragt, ob sie die akute Phase als „sehr angenehm“, „erträglich“ oder „schrecklich“ empfanden, da es in der Literatur Hinweise darauf gibt, dass einige PatientInnen mit Katatonie von eher angenehmen Gefühlen während der katatonen Phase berichten [11].

Für die Datenanalyse wurden 24 Symptome (Items #1 bis #24) berücksichtigt, die auf einer 3‑stufigen Likert-Skala mit Werten von 0 bis 2 bewertet wurden (0 = fehlende Veränderung/Abnormalität, 1 = Veränderung/Abnormalität definitiv vorhanden, aber mäßig und gelegentlich vorhanden mit der Möglichkeit einer Unterbrechung, 2 = Veränderung/Abnormalität ständig und gravierend vorhanden ohne Möglichkeit einer Unterbrechung). Der Gesamtscore kann von 0 bis 48 Punkten variieren.

Für die weitere Analyse wurden nur PatientInnen berücksichtigt, die sich zumindest teilweise an die akute katatone Phase erinnern konnten. Demografische und klinische Charakteristika der Studienpopulation (*n* = 46) sind in Tab. 1 aufgeführt. Für die Reliabilitätsanalyse wurde Cronbach’s Alpha berechnet, um die interne Konsistenz der NSSC zu untersuchen. Cronbach’s Alpha war 0,91 für die gesamte NSSC mit 24 Items. Es bestand eine signifikante positive Korrelation zwischen der NSSC und psychopathologischen Symptomen (NCRS, Positive and Negative Syndrome Scale [PANSS], Brief Psychiatric Rating Scale [BPRS] und Trait Ängstlichkeit, gemessen mit dem State Trait Anxiety Inventory [STAI]; siehe Tab. 2). Es bestand kein signifikanter Zusammenhang zwischen dem NSSC-Gesamtscore und dem Gesamtscore der Bush-Francis Catatonia Rating Scale (BFCRS; *r* = 0,24, *p* = 0,11) und dem Global Assessment of Functioning (GAF; *r* = −0,16; *p* = 0,30), Emotionsregulation (Emotion Regulation Questionnaire [ERQ], Subskala Suppression [*r* = 0,05; *p* = 0,75], Subskala Reappraisal [*r* = 0,11; *p* = 0,84]) sowie den Subskalen des Childhood-Trauma-Questionnaires (CTQ) (alle *p* > 0,05). Die NSSC war positiv korreliert mit Trait-Ängstlichkeit (*r* = 0,64; *p* < 0,01), jedoch nicht mit Einsamkeitserleben (*r* = 0,09; *p* = 0,59).

**Table 1:** 

Variable	Mittelwert	Standardabweichung
Alter	38,02	14,69
Geschlecht (m/w)	31/16	–
Bildungsjahre	12,89	2,76
Olanzapinäquivalente	14,04	8,21
*NSSC gesamt*	15,04	9,32
*NSSC motorisch*	2,22	2,78
*NSSC affektiv*	6,83	3,68
*NSSC behavioral*	3,78	2,99
*NCRS gesamt*	10,78	4,90
*NCRS motorisch*	2,50	2,93
*NCRS affektiv*	4,65	2,51
*NCRS behavioral*	3,63	1,82
BFCRS gesamt	3,04	2,35
*PANSS gesamt*	70,59	14,99
*PANSS positiv*	14,72	6,21
*PANSS negativ*	19,46	6,59
*PANSS generell*	36,41	7,21
*GAF*	42,72	10,71
*STAI gesamt*	51,53	12,13
*UCLA gesamt*	53,00	9,26
*ERQ reappraisal*	21,64	7,99
*ERQ suppression*	14,93	4,68
*CTQ emotionale Misshandlung*	11,14	5,53
*CTQ körperliche Misshandlung*	7,42	3,63
*CTQ sexueller Missbrauch*	6,67	3,47
*CTQ emotionale Vernachlässigung*	13,11	5,77
*CTQ körperliche Vernachlässigung*	8,89	3,60
*CTQ Bagatellisierung*	0,29	0,62

*NSSC* Northoff Skala für subjektives Erleben bei Katatonie, deutsche Version; *NCRS* Northoff Catatonia Rating Scale; *PANSS* Positive and Negative Syndrome Scale; *BFCRS* Bush Francis Catatonia Rating Scale; *BPRS* Brief Psychiatric Rating Scale; *GAF* Global Assessment of Functioning; *STAI* State-trait anxiety inventory, Subskala Trait-Ängstlichkeit, *UCLA* Loneliness scale, *ERQ* Emotion Regulation Questionnaire (*r* reappraisal, *s* suppression); *CTQ* Childhood Trauma Questionnaire (*em* emotionale Misshandlung, *km* körperliche Misshandlung, *sm* sexueller Missbrauch, *ev* emotionale Vernachlässigung, *kv* körperliche Vernachlässigung, *b* Bagatellisierung)

**Table 2:** 

Variable	NSSC t	NSSC m	NSSC a	NSSC b	NCRS t	NCRS m	NCRS a	NCRS b	BFCRS t	PANSS t	PANSS p	PANSS n	PANSS g	BPRS	GAF	STAI	UCLA I	ERQ r	ERQ s	CTQ em	CTQ km	CTQ sm	CTQ ev	CTQ kv	CTQ b
NSSC t	–	–	–	–	–	–	–	–	–	–	–	–	–	–	–	–	–	–	–	–	–	–	–	–	–
NSSC m	0,83**	–	–	–	–	–	–	–	–	–	–	–	–	–	–	–	–	–	–	–	–	–	–	–	–
NSSC a	0,92**	0,67**	–	–	–	–	–	–	–	–	–	–	–	–	–	–	–	–	–	–	–	–	–	–	–
NSSC b	0,88**	0,69**	0,72**	–	–	–	–	–	–	–	–	–	–	–	–	–	–	–	–	–	–	–	–	–	–
NCRS t	0,43**	0,46**	0,37**	0,36*	–	–	–	–	–	–	–	–	–	–	–	–	–	–	–	–	–	–	–	–	–
NCRS m	0,09	0,19	0,03	0,01	0,68**	–	–	–	–	–	–	–	–	–	–	–	–	–	–	–	–	–	–	–	–
NCRS a	0,50**	0,48**	0,46**	0,49**	0,60**	−0,08	–	–	–	–	–	–	–	–	–	–	–	–	–	–	–	–	–	–	–
NCRS b	0,34**	0,26	0,32*	0,27*	0,76**	0,34*	0,37*	–	–	–	–	–	–	–	–	–	–	–	–	–	–	–	–	–	–
BFCRS t	0,24	0,35*	0,12	0,31*	0,19	−0,05	0,28	0,20	–	–	–	–	–	–	–	–	–	–	–	–	–	–	–	–	–
PANSS t	0,30*	0,36*	0,16	0,39*	0,47**	0,31*	0,37*	0,27	0,23	–	–	–	–	–	–	–	–	–	–	–	–	–	–	–	–
PANSS p	0,09	0,30	−0,06	0,19	0,344**	0,38**	0,31*	0,16	0,20	0,71**	–	–	–	–	–	–	–	–	–	–	–	–	–	–	–
PANSS n	0,20	0,13	0,09	0,28	0,13	0,12	0,02	0,12	0,10	0,70**	0,18	–	–	–	–	–	–	–	–	–	–	–	–	–	–
PANSS g	.**	0,38*	0,29*	0,39**	0,49**	0,21	0,48**	0,32*	0,20	0,83**	0,46**	0,37*	–	–	–	–	–	–	–	–	–	–	–	–	–
BPRS	0,33*	0,40*	0,22	0,34*	0,52**	0,33*	0,41**	0,35*	0,23	0,90**	0,68**	0,45**	0,90**	–	–	–	–	–	–	–	–	–	–	–	–
GAF	−0,04	−0,02	−0,02	−0,10	−0,25	−0,06	−0,23	−0,26	−0,016	−0,44*	−0,29*	−0,37*	−0,32	−0,47*	–	–	–	–	–	–	–	–	–	–	–
STAI	0,64**	0,46**	0,70**	0,46**	0,32	0,04	0,34*	0,35*	0,04	0,01	−0,15	−0,13	0,27	0,11	0,25	–	–	–	–	–	–	–	–	–	–
UCLA	0,11	0,02	0,10	0,15	−0,04	−0,23	0,29	−0,12	0,09	0,17	0,01	0,10	0,24	0,20	0,04	0,31	–	–	–	–	–	–	–	–	–
ERQ r	−0,05	0,03	−0,12	0,05	0,02	0,06	0,08	−0,14	0,11	0,29	0,42*	−0,09	0,32	0,33*	−0,20	−0,15	−0,10	–	–	–	–	–	–	–	–
ERQ s	−0,01	−0,18	0,00	0,13	0,09	0,06	0,09	0,04	0,05	0,07	0,07	−0,17	0,24*	0,10	0,15	0,28	0,24	0,18	–	–	–	–	–	–	–
CTQ em	0,03	−0,13	0,14	−0,07	0,10	0,07	0,08	0,06	−0,13	0,06	−0,11	−0,07	0,29	0,24	0,07	0,24	0,34*	0,07	0,41*	–	–	–	–	–	–
CTQ km	0,01	0,06	0,02	−0,05	0,09	0,07	0,13	−0,06	−0,15	−0,05	0,01	−0,26	0,12	−0,05	0,20	0,05	0,34*	0,04	0,13	0,57**	–	–	–	–	–
CTQ sm	0,18	0,07	0,22	0,14	−0,03	−0,08	−0,09	0,19	0,00	−0,03	−0,11	−0,205	0,08	0,01	0,08	0,27	−0,08	0,05	0,14	0,25	0,04	–	–	–	–
CTQ ev	−0,17	−0,29	−0,11	−0.16	−0,24	−0,10	−0,16	−0,29	−0,29	−0,17	−0,26	−0,19	0,04	−0,09	0,14	0,10	0,46*	−0,04	0,49**	0,60**	0,42*	0,20	–	–	–
CTQ kv	0,03	−0,09	0,07	0,10	−0,02	−0,03	−0,00	0,01	0,12	0,11	0,10	−0,05	0,19	0,14	0,16	0,13	41**	−0,05	0,53**	0,59**	0,42**	0,25*	0,65**	–	–
CTQ b	0,25	0,53**	0,17	0,12	0,20	0,14	0,26	−0,02	0,40*	0,11	0,18	0,07	0,02	0,07	−0,03	−0,07	−0,30	0,08	−0,26	−0,27	−0,07	−0.11	−0,37	−0,19	–

Korrelationstabelle. Pearson-Korrelationskoeffizenten (r-Werte, zweiseitig getestet, *p < 0,05; **p < 0,01)

*NSSC* Northoff Skala für subjektives Erleben bei Katatonie, deutsche Version (*t* gesamt, *m* motor, *a* affektiv, *b* behavioral); *NCRS* Northoff Catatonia Rating Scale (*t* gesamt, *m* motor, *a* affektiv, *b* behavioral); *PANSS* Positive and Negative Symptoms Scale (*t* gesamt, *p* positiv, *n* negativ, *g* generell); *BFCRS* Bush Francis Catatonia Rating Scale; *BPRS* Brief Psychiatric Rating Scale; *GAF* Global Assessment of Functioning; *STAI* State-Trait Anxiety Inventory, Subskala Trait-Ängstlichkeit; *UCLA* UCLA Loneliness Scale; *ERQ* Emotion Regulation Questionnaire (*r* reappraisal; *s* suppression); *CTQ* Childhood Trauma Questionnaire (*em* emotionale Misshandlung, *km* körperliche Misshandlung, *sm* sexueller Missbrauch, *ev* emotionale Vernachlässigung, *kv* körperliche Vernachlässigung, *b* Bagatellisierung)

## Diskussion

Das Hauptziel dieser Studie war die Erweiterung und Validierung des ersten klinischen Instruments im deutschsprachigen Raum zur spezifischen Erfassung des subjektiven Erlebens bei PatientInnen mit Katatonie. Diese Studie konnte drei Hauptergebnisse erzielen: *Erstens* haben wir die ursprüngliche deutsche Version der NSSC von 1996 [11] modifiziert und erweitert. *Zweitens* zeigte die erweiterte Version von NSSC eine hervorragende interne Konsistenz. *Drittens* wies die NSSC signifikante positive Korrelationen mit psychopathologischen Symptomen, die anhand etablierter klinischer Skalen bei Katatonie erhoben wurden, auf.

Bisher haben nur wenige Kliniker- und ForscherInnen das subjektive Erleben bei Katatonie untersucht. Neben der Studie von Northoff et al. [11] wurde lediglich in einer neueren Studie von Zingela et al. [30] das subjektive Erleben bei Katatonie in einem qualitativen Ansatz untersucht. Zingela et al. [30] zeigten, dass das subjektive Erleben von PatientInnen mit Katatonie hauptsächlich durch überwältigende Ängste und depressive Symptome gekennzeichnet ist. Die AutorInnen konnten die früheren Ergebnisse von Northoff et al. [11] replizieren und schlussfolgerten, dass überwältigende Emotionen zum sozialen Rückzug katatoner PatientInnen führen. Darüber hinaus haben die AutorInnen auch die Notwendigkeit psychotherapeutischer und psychosozialer Interventionen bei PatientInnen mit Katatonie betont. Aus klinischer Praxis und der Literatur ist bekannt, dass intensive personelle Begleitung und psychotherapeutische Strategien notwendig sind, um PatientInnen z. B. mit Negativismus von der Einnahme von Flüssigkeit, Nahrung und Medikamenten zu überzeugen, bei häufig gleichzeitig starken Furchtaffekten. Heckers und Walther [31] weisen darauf hin, dass insbesondere ein flexibles Behandlungskonzept PatientInnen mit Negativismus oder Ambitendenz nützen könnte, in welchem insbesondere Schwierigkeiten bei der Alltagsbewältigung Hinweise auf wiederkehrende katatone Episoden liefern können und Strategien zur Rückfallprophylaxe abgeleitet werden können. Aus der klinischen Erfahrung der AutorInnen ist es insbesondere im postakuten Behandlungssetting notwendig, durch kontinuierliche Gesprächsangebote und gezielte psychotherapeutische Interventionen die PatientInnen bei der Restitution kognitiver und motorischer Fähigkeiten, der Neuinterpretation negativer Stimuli, der Etablierung einer Tagesstruktur und der Wiederaufnahme sozialer Kontakte zu unterstützen. Dies könnte durch einen prozessorientierten kognitiven verhaltenstherapeutischen Ansatz gelingen, in welchem passgenau für die PatientInnen domänenbasierte Behandlungsfoci abgeleitet werden (Kognition, Affekt, Selbst, Verhalten, Motivation, Biophysiologie, Kontext, soziokulturelle Faktoren; [32]). Ein großer Vorteil dieses Ansatzes könnte in der transdiagnostischen Anwendung bestehen, da Katatonie in der ICD-11 auch als eigenständige Entität in Verbindung mit anderen psychischen Störungen diagnostiziert werden kann. In einer weiteren aktuellen Studie von Dawkins et al. [8] wurde ein narrativer Ansatz genutzt, um das subjektive Erleben von Katatonie zu untersuchen. Die AutorInnen durchsuchten hierfür Gesundheitsakten von PatientInnen mit Katatonie. Anhand der Analyse von 1456 validierten Katatoniediagnosen zeigten die AutorInnen, dass es drei klinische Subtypen der Katatonie gibt, nämlich parakinetische, hypokinetische und absetzassoziierte Katatonie. Diese Studie konnte 68 PatientInnen mit subjektiven Aussagen über die Zeit während der katatonen Episode identifizieren. Von diesen PatientInnen berichteten 35 % über massive Ängste, wobei die Mehrheit (72 %) dieser PatientInnen eine plausible Erklärung (z. B. Halluzinationen, Wahnvorstellungen und nichtpsychotische Beschwerden) für die katatonen Symptome lieferte. Dies steht auch im Einklang mit der klinischen Erfahrung der AutorInnen, dass die Mehrheit der PatientInnen eine subjektiv plausible Erklärung für ihre Akinese oder Hyperkinese hat. Insgesamt zeigen die beiden genannten Studien, wie wichtig die Erfassung subjektiver Symptome bei Katatonie ist, um über die medikamentöse Behandlung hinaus (psycho-)therapeutische Optionen für diese oftmals schwer kranke PatientInnengruppe zu etablieren.

Um der derzeit noch geringen Evidenz zum subjektiven Erleben von Katatonie zu begegnen, haben wir neben der Verwendung der Likert-Skala für jedes Item der NSSC auch möglichst viele der 40 Symptome der NCRS in die NSSC übernommen. Die Modifikation und Erweiterung der ursprünglichen NSSC war somit erfolgreich und die Skala fand bei KollegInnen und Studierenden großen Anklang. Darüber hinaus wurden die ersten 46 PatientInnen mit Katatonie gemäß ICD-11 mit der NSSC untersucht. Die NSSC korrelierte signifikant mit dem Gesamtwert der NCRS. Interessanterweise fanden wir keine Korrelation mit der motorischen Unterskala der NCRS. Dies deckt sich allerdings mit den Ergebnissen von Northoff et al. [11], dass 50 % der katatonen PatientInnen keine subjektiven Bewegungseinschränkungen während ihrer katatonen Zustände berichteten. Die AutorInnen begründeten dies durch eine mögliche Differenzierung der Gruppe der PatientInnen mit Katatonie in einen affektiven und einen nichtaffektiven Subtyp der Katatonie. Der zweitgenannte Subtypus ist eher durch starke Ambivalenz und Blockade des Willens charakterisiert. Eine andere Erklärung ist, dass die PatientInnen in der vorliegenden Studie während der Teilremission akuter katatoner Symptomatik untersucht wurden und daher keine starken Auffälligkeiten mehr im Bereich der Motorik aufwiesen. Diese Erklärung ist jedoch nicht plausibel, da sich in den objektiven Messinstrumenten (NCRS, BFCS etc.) weiterhin deutliche Auffälligkeiten der Motorik zeigten und alle 46 PatientInnen zum Zeitpunkt der Studienuntersuchung die Kriterien für Katatonie gemäß ICD-11 erfüllten. Aus Erfahrung der AutorInnen ist der Kontrast zwischen Eigen- und Fremdwahrnehmung ein Charakteristikum für das akute katatone Zustandsbild. Oft berichten PatientInnen im Anschluss an eine akute Episode, in der sie regungslos in einer Pose verharrten und mutistisch waren, dass sie diesen Zustand jederzeit hätten verlassen können. Dies steht in starkem Kontrast zum klinischen Untersuchungsbefund der UntersucherInnen. Der erhöhte Muskeltonus und die motorischen Einschränkungen sind vorhanden und können häufig nur medikamentös behandelt werden. Hierin zeigt sich auch ein Unterschied zu Morbus Parkinson oder anderen neurologischen Bewegungsstörungen, bei denen den PatientInnen die eigenen motorischen Defizite durchgehend bewusst sind und diese als unangenehm berichten. Weiterhin unterstützt dieser Befund das psychomotorische Konzept der Katatonie im Vergleich zu einem rein motorischen Konzept. Instrumente wie der CS von Dell’Osso et al. [24] können eine hilfreiche Ergänzung zur NSSC darstellen um das gesamte Katatoniespektrum mit einzubeziehen. Zusammenfassend lässt sich sagen, dass in der vorliegenden Arbeit die Ergebnisse von Northoff et al. [11] repliziert werden konnten.

Obwohl die PatientInnen mit Katatonie in unserer Stichprobe im Mittel starke Beeinträchtigungen in mehreren Bereichen des allgemeinen Funktionsniveaus aufwiesen, fanden wir interessanterweise keine signifikante Korrelation zwischen dem NSSC-Gesamtwert und dem GAF-Wert. Ähnliche Befunde wurden in anderen Arbeiten zum subjektiven Erleben, wie beispielsweise dem subjektiven Erleben wahrgenommener Stigmatisierung bei PatientInnen mit Schizophrenie, berichtet [33]. In der Arbeit von Zäske et al. [33] zeigten sich keine Korrelationen der wahrgenommenen Stigmatisierung mit klinischen Skalen sowie dem GAF-Wert. Eine mögliche Begründung könnte darin liegen, dass das Globale Funktionsniveau durch ein objektives Rating von erfahrenen KlinikerInnen erfasst wird, es sich aber bei der Einschätzung des subjektiven Erlebens der Katatonie um einen Selbstauskunftsfragebogen handelt. Des Weiteren ist es möglich, dass die NSSC spezifische Aspekte des inneren Erlebens erfasst, die nicht zwingend mit dem Globalen Funktionsniveau der Person in Beziehung stehen müssen, welches verschiedene Lebensbereiche wie Arbeit, soziale Funktionen und das Aktivitätsniveau mitberücksichtigt [34]. Überraschenderweise fanden wir keine Zusammenhänge zwischen dem subjektiven Erleben der Katatonie und objektiv gemessenen katatonen Symptomen mit der Bush-Francis Catatonia Rating Scale. Dies könnte in Einklang stehen mit den bereits erwähnten Befunden von Northoff et al. [11], dass ein Subtyp der PatientInnen mit Katatonie keine Erinnerung an motorische Symptome der Katatonie aufweist. Bei der BFCRS handelt es sich um das meist verwendete Instrument zur Objektivierung katatoner Symptome [35], berücksichtigt allerdings überwiegend motorische Phänomene. Die NCRS ist bislang die einzige Ratingskala zur Einschätzung der Katatonie, in welcher auch explizit affektive Symptome berücksichtigt werden [36]. Wir fanden keine Zusammenhänge zwischen dem subjektiven Erleben bei Katatonie mit Subskalen der Emotionsregulation, gemessen mit dem ERQ. Das ist ein möglicher Hinweis darauf, dass aufgrund einer veränderten Verarbeitung negativer Stimuli [1] katatone PatientInnen weniger explizite Emotionsregulationsstrategien wie Unterdrückung negativer Emotionen und positive Neubewertung anwenden. Signifikante Zusammenhänge zwischen dem NSSC-Gesamtscore und den Subskalen des CTQ konnten ebenfalls nicht identifiziert werden. Interessanterweise korrelierte die Subskala Bagatellisierung des CTQ (CTQb) mit der motorischen Subskala der NSSC (Tab. 2, NSSCm), der motorischen Subskala der NCRS (NCRS m) und der stark auf motorische Symptome der Katatonie ausgerichteten BFCRS. Je höher die motorischen Symptome der Katatonie ausgeprägt waren, sowohl in der subjektiven als auch objektiven Einschätzung, desto höher waren die Werte innerhalb der Bagatellisierungssubskala des CTQ. Höhere Werte in der CTQb geben Hinweise darauf, dass eine Antworttendenz zur Bagatellisierung traumatischer Ereignisse vorliegen könnte [37] und die PatientInnen während der akuten Katatonie nicht in der Lage sind, sich mit ihren früheren traumatischen oder belastenden Lebensereignissen auseinanderzusetzen. Möglicherweise steht dies im Zusammenhang mit veränderter emotionaler Reizverarbeitung und der Dysfunktion im orbitofrontalen Kortex [38] und der Amygdala [39].

## Stärken und Limitationen

Die vorliegende Arbeit leistet einen Beitrag, im deutschsprachigen Raum nähere Einblicke in die subjektive Erlebenswelt von Menschen mit Katatonie zu erlangen. Die NSSC-dv stellt somit ein nützliches Tool für KlinikerInnen, ForscherInnen sowie auch möglicherweise Angehörige, die während akut katatoner Zustände keinen Zugang zu den Betroffenen fanden, dar. Langfristig könnte es über den Zugang zum subjektiven Erleben der PatientInnen gelingen, bedürfnisorientierte psychotherapeutische Interventionen zu entwickeln, damit die PatientInnen nach Teilremission lebensbedrohlicher Symptome einen besseren Umgang mit noch bestehender Symptomatik erlernen. Hervorzuheben ist weiterhin die Anwendung der erst kürzlich veröffentlichten Diagnosekriterien der ICD-11 in Verbindung mit gängigen Ratingskalen zur Einschätzung der katatonen Symptomatik. Die vorliegende Arbeit verfolgte einen transdiagnostischen Ansatz, der den diagnostischen Änderungen in der ICD-11 Rechnung trägt, dass Katatonie als eigenständige Erkrankung diagnostiziert werden kann.

Neben Stärken sind auch Limitationen dieser Studie zu beachten: Durch die Anwendung der diagnostischen Kriterien der ICD-11 unterscheidet die vorliegende Arbeit nicht explizit zwischen PatientInnen mit Schizophreniespektrumstörungen (Schizophrenie, schizoaffektive Störung, akute polymorphe psychotische Störung, schizotype Störung) und affektiven Störungen, welche sich möglicherweise in ihrer Beantwortung der NSSC voneinander unterscheiden [5]. Psychopharmakologischer Behandlungsstandard der Katatonie stellt bislang die Behandlung mit Benzodiazepinen dar. Medikamenteneffekte müssen deshalb auch in zukünftigen Studien zusätzlich zu den Kovariaten Alter, Geschlecht, Bildungsjahren und Dauer der Erkrankung mitberücksichtigt werden, da möglicherweise die anxiolytische Medikation affektive Symptome maskiert, welche für ein besseres Verständnis des subjektiven Erlebens bei Katatonie essenziell sind. Aufgrund der aktuell noch kleinen Stichprobe haben wir die Faktorenstruktur nicht näher untersucht, da die Güte der Faktorenstruktur von der Stichprobengröße (Stichprobengröße von mindestens *n* = 60 notwendig) abhängig ist [40]. Nicht zuletzt handelt es sich bei der vorgestellten Arbeit um ein Querschnittsdesign. Die übergeordnete whiteCAT-Studie ist als prospektives Studiendesign konzipiert (nähere Angaben s. [29]), in diesem Rahmen wird die NSSC zu drei Messzeitpunkten (V1, V2 + 6 Wochen, V3 + 12 Wochen) erhoben, sodass langfristig längsschnittliche Daten der NSSC zur Verfügung gestellt werden können.

## Fazit für die Praxis

Die modifizierte und validierte NSSC ermöglicht KlinikerInnen und ForscherInnen, einen systematischen Einblick in das subjektive Erleben von PatientInnen mit Katatonie zu bekommen. Diese Studie unterstreicht das Potenzial, mehr Erkenntnisse über das subjektive Erleben während katatoner Zustände zu gewinnen, um maßgeschneiderte Behandlungsmöglichkeiten für die Bedürfnisse der betroffenen PatientInnen zu entwickeln.

### Supplementary Information





