# Supplementary material for: German version of the Northoff scale for subjective experience in catatonia (NSSC-dv): A validated instrument for examination of the subjective experience in catatonia
Source: Nervenarzt. 2023 Dec 13;95(1):10–7. [Article in German] doi: 10.1007/s00115-023-01575-4 (PMC10808566; doi:10.1007/s00115-023-01575-4)
Supplement: Supplementary file 1 [file 115_2023_1575_MOESM1_ESM.docx]

**Supplement**

*Studienpopulation*

Ausschlusskriterien waren: (1) Alter <18 oder >64 Jahre; (2) Vorgeschichte eines Hirntraumas oder einer neurologischen Erkrankung (insbesondere Bewegungsstörungen, z.B. Morbus Parkinson); (3) Alkohol-/Substanzabhängigkeit innerhalb von 12 Monaten vor der Studienteilnahme; oder (4) MRT-Kontraindikationen. Erkrankungen, die möglicherweise die Funktion des Zentralnervensystems beeinträchtigen, sowie Herz-Kreislauf- oder Stoffwechselerkrankungen bei PatientInnen mit Katatonie wurden durch körperliche Untersuchung, Laborkontrolle, EKG, EEG und MRT ausgeschlossen. Darüber hinaus wurden bei keiner/m der PatientInnen am Tag der Untersuchung schwerwiegende Komplikationen der Katatonie, wie Dehydrierung oder Elektrolytstörungen, festgestellt. Die örtliche Ethikkommission (Medizinische Fakultät der Universität Heidelberg, Deutschland) hat die Studie genehmigt (Studiennummer: 2021-613). Nachdem alle Ziele und Abläufe der Studie vollständig erläutert wurden, haben wir von allen StudieninteressentInnen eine schriftliche Einverständniserklärung eingeholt.

*Erweiterung der ursprünglichen Northoff‘schen Selbstbeurteilungsskala*

Wir haben in folgenden Schritten die Northoff‘sche Selbstbeurteilungsskala erweitert: Erstens haben wir das Bewertungssystem verändert und eine neue Bewertung auf der Grundlage einer Likert-Skala für jedes Item entwickelt, weil die ursprüngliche Version nicht die gesamte Breite des Schweregrads des subjektiven Erlebens abbilden konnte. Hierbei haben wir uns an der dreistufigen Likert-Skala orientiert, welche auch im Rahmen der NCRS verwendet wird, zur besseren Vergleichbarkeit der Instrumente. Zweitens führten wir eine systematische Recherche in PubMed mit den Begriffen „Katatonie“ und „subjektiv“ oder „Subjektivität“ durch, um Studien zum subjektiven Erleben von PatientInnen mit Katatonie zu identifizieren und die einzelnen Bewertungsmöglichkeiten zu bestimmen. Drittens haben wir (GAB, SF und DH) eine Suche innerhalb der NCRS [1], BFCRS [2] und ICD-11 durchgeführt, um zusätzliche katatone Symptome, welche das gesamte Spektrum des subjektiven Erlebens von PatientInnen mit Katatonie abbilden (https://icd.who.int/browse11), zu identifizieren. Abschließend haben wir die neu ergänzten Symptome mit ExpertInnen auf diesem Gebiet (GN, KMK und RCW) diskutiert, um sicherzustellen, dass sie klinisch relevant, prägnant und gut in der Praxis anwendbar sind.

*Klinische Untersuchung*

Alle PatientInnen wurden während einer stationären oder ambulanten Behandlung nach teilweiser Remission akuter psychopathologischer Symptome am Zentralinstitut für Seelische Gesundheit (CIMH) in Mannheim untersucht. Bei allen inkludierten PatientInnen wurde eine Katatonie (Katatonie in Verbindung mit einer anderen psychischen Störung – 6A40) gemäß ICD-11 diagnostiziert. In der ICD-11 sind für die Diagnose einer Katatonie drei oder mehr der folgenden Symptome in einem der drei Bereiche (verminderte, erhöhte oder abnormale psychomotorische Aktivität) erforderlich. Zu beachten ist, dass bei Vorliegen von mehr als einem Symptom innerhalb der erhöhten psychomotorischen Aktivität diese nur als jeweils ein Punkt gewertet werden. Der Schweregrad der katatonen Symptome wurde mit der Deutschen Version der NCRS (NCRS_dv) erfasst [3]. Das subjektive Erleben wurde mit der modifizierten und erweiterten deutschen Version der NSSC erhoben. PatientInnen mit Katatonie sollten angeben, wie sie ihre katatonen Symptome derzeit erleben oder in den letzten 7 Tagen erlebt haben. Dieser Zeitraum wurde gewählt, damit sich die PatientInnen so genau wie möglich an ihre Symptome erinnern können und eine Verzerrung in Bezug auf NCRS und BFCRS vermieden werden kann. Wir haben bewusst keinen längeren Zeitraum, z. B. 14 Tage, gewählt, da sich die PatientInnen möglicherweise nicht mehr genau an ihre Symptome erinnern und dies zu einer möglichen Verzerrung in Bezug auf NCRS oder BFCRS führen würde. Die Bewertung der allgemeinen psychopathologischen Symptome und der globalen Funktionsfähigkeit erfolgte mit der Positive and Negative Syndrome Scale [PANSS] [4], Brief Psychiatric Rating Scale [BPRS] [5] und Global Assessment of Functioning [GAF] [6]. Angstsymptome wurden mit der Subskala Trait-Angst des Stait-Trait-Angst-Inventars [STAI] [7] und das Einsamkeitserleben mit der deutschen Version der UCLA-Einsamkeitsskala [UCLA] erfasst [8]. Weiterhin untersucht wurden Emotionsregulationsstrategien mit dem Emotionsregulationsfragebogen [ERQ] sowie das Vorliegen von traumatischen Erfahrungen in Kindheit und Jugend gemessen mit dem Childhood Trauma Questionnaire [CTQ].

*Statistische Analyse*

Zunächst wurde eine deskriptive Analyse der demografischen und klinischen Daten bei katatonen PatientInnen (Tabelle 1) durchgeführt. Um die Zuverlässigkeit auf Symptomebene zu bewerten, wurde Cronbach‘s Alpha gemäß SPSS Version 26 berechnet. Zur Berechnung der Kriterium- und Divergenzvalidität wurde eine Pearson-Korrelation (zweiseitig) durchgeführt, um die Beziehung zwischen NSSC und psychopathologischen Symptomen (NCRS, BFCRS, PANSS, STAI Subskala Trait-Ängstlichkeit und BPRS), Einsamkeitserleben (UCLA Loneliness Scale) und globalem Funktionsniveau (GAF) zu bestimmen. Außerdem untersuchten wir mittels Pearson-Korrelation (zweiseitig) den Zusammenhang der NSSC und den Emotionsregulationsstrategien Suppression und positive Neubewertung (ERQ) sowie mit verschiedenen Bereichen aversiver Kindheitserlebnisse und traumatischer Lebensereignisse (CTQ) [9]. Die CTQ Subskalen emotionale Misshandlung, körperliche Misshandlung, sexueller Missbrauch, emotionale Vernachnässigung, körperliche Vernachlässigung sowie die Bagetellisierung aversiver Kindheitserlebnisse wurden näher untersucht.

**Referenzen**

1. Northoff, G., et al., *Catatonia as a psychomotor syndrome: a rating scale and extrapyramidal motor symptoms.* Mov Disord, 1999. **14**(3): p. 404-16.

2. Bush, G., et al., *Catatonia. I. Rating scale and standardized examination.* Acta Psychiatr Scand, 1996. **93**(2): p. 129-36.

3. Hirjak, D., et al., *[German version of the Northoff catatonia rating scale (NCRS-dv) : A validated instrument for measuring catatonic symptoms].* Nervenarzt, 2017. **88**(7): p. 787-796.

4. Kay, S.R., A. Fiszbein, and L.A. Opler, *The positive and negative syndrome scale (PANSS) for schizophrenia.* Schizophr Bull, 1987. **13**(2): p. 261-76.

5. Andersen, J., et al., *The Brief Psychiatric Rating Scale: Schizophrenia, Reliability and Validity Studies.* Nordisk Psykiatrisk Tidsskrift., 1986. **40:2.**: p. 135-138.

6. DSM-III.R., D.K.u.D.d.d.u.s.M.p.S.r., *GAF-Skala: Global Assessment of Functioning Scale.* 1989, Beltz: Weinheim, Basel.

7. Spielberger, C.D., et al., *Das State-Trait-Angstinventar: STAI.* 2001: Göttingen, Germany: Beltz Test.

8. Döring, N. and J. Bortz, *Psychometrische Einsamkeitsforschung: Deutsche Neukonstruktion der UCLA Loneliness Scale.* Diagnostica., 1993.

9. Klinitzke, G., et al., *[The German Version of the Childhood Trauma Questionnaire (CTQ): psychometric characteristics in a representative sample of the general population].* Psychother Psychosom Med Psychol, 2012. **62**(2): p. 47-51.
