# Supplementary material for: German version of the Northoff scale for subjective experience in catatonia (NSSC-dv): A validated instrument for examination of the subjective experience in catatonia
Source: Nervenarzt. 2023 Dec 13;95(1):10–7. [Article in German] doi: 10.1007/s00115-023-01575-4 (PMC10808566; doi:10.1007/s00115-023-01575-4)
Supplement: Supplementary file 2 [file 115_2023_1575_MOESM2_ESM.docx]

**Anhang.**

**Northoff Scale for Subjective Experience in Catatonia (NSSC) – deutsche Version**

**Patient ID:_______________**

**Datum:___________________**

**Northoff Scale for Subjective Experience in Catatonia (NSSC)**

Dieser Fragebogen dient dazu, Ihre innere Verfassung in der Zeit zu beschreiben, in der andere Menschen keinen richtigen Kontakt zu Ihnen fanden. Bitte kreuzen Sie bei den unten aufgeführten Fragen Ihre eigene Auffassung an.

0 = fehlende Abnormalitäten/Veränderungen.

1 = Veränderung/Abnormalität definitiv vorhanden, aber mäßig und gelegentlich vorhanden mit der Möglichkeit einer Unterbrechung.

2 = Veränderung/Abnormalität ständig und gravierend vorhanden ohne Möglichkeit einer Unterbrechung.

Bevor Sie mit der Beantwortung der Fragen beginnen, überlegen Sie wie gut Sie sich an die Zeit des katatonen Zustandes erinnern können. Nur wenn Sie „sehr gut“ oder „teilweise“ ankreuzen, ist es sinnvoll mit der Beantwortung der anderen Fragen weiterzumachen.

**Ich erinnere mich an diese Zeit:**

0🞎 Sehr gut

1🞎 Teilweise

2🞎 Gar nicht

**1 - Bewegungen ausführen**

0🞎 Ich hatte keine Probleme, Bewegungen auszuführen.

1🞎 Ich hatte definitiv Probleme, Bewegungen auszuführen, allerdings nur mäßig und gelegentlich.

2🞎 Ich konnte keine Bewegung mehr ausführen.

**2 - Wille**

0🞎 Mein Wille war ganz frei und nicht blockiert/beeinflusst.

1🞎 Mein Wille war definitiv blockiert, allerdings nur mäßig und gelegentlich.

2🞎 Mein Wille war total blockiert/beeinflusst.

**3 - Sprachliche Probleme**

0🞎 Ich hatte keine Probleme, mich sprachlich zu äußern.

1🞎 Ich hatte definitiv Probleme, mich sprachlich zu äußern, allerdings nur mäßig und gelegentlich.

2🞎 Ich konnte kein Wort mehr herausbringen.

**4 - Ideen motorisch umsetzen**

0🞎 Ich konnte meine Ideen motorisch umzusetzen.

1🞎 Ich hatte definitiv Probleme, meine Ideen motorisch umzusetzen, allerdings nur mäßig und gelegentlich.

2🞎 Ich konnte meine Ideen überhaupt nicht motorisch umsetzen.

**5 - Freude**

0🞎 Ich hatte intensive Gefühle der Freude.

1🞎 Ich hatte definitiv Gefühle der Freude, allerdings nur mäßig und gelegentlich.

2🞎 Ich hatte keine Gefühle der Freude.

**6 - Angst**

0🞎 Ich hatte keine Angst.

1🞎 Ich hatte definitiv Gefühle der Angst, allerdings nur mäßig und gelegentlich.

2🞎 Ich hatte intensive Gefühle der Angst.

**7 - Gefühle**

0🞎 Diese Gefühle störten mich nicht.

1🞎 Diese Gefühle haben mich definitiv gestört, allerdings nur mäßig und gelegentlich.

2🞎 Diese Gefühle haben mich völlig überwältigt und blockiert.

**8 - Bewegungsunfähigkeit**

0🞎 Diese Gefühle machten mich nicht bewegungsunfähig.

1🞎 Diese Gefühle machten mich definitiv bewegungsunfähig, allerdings nur mäßig und gelegentlich.

2🞎 Diese Gefühle machten mich völlig steif und bewegungslos.

**9 - Kontrolle über Emotionen**

0🞎 Ich hatte volle Kontrolle über meine Gefühle.

1🞎 Die Kontrolle über meine Gefühle war definitiv beeinträchtigt, allerdings nur mäßig und gelegentlich.

2🞎 Ich hatte keinerlei Kontrolle und Einfluss auf meine Gefühle.

**10 - Kontrolle über Gedanken**

0🞎 Ich hatte volle Kontrolle über meine Gedanken.

1🞎 Die Kontrolle über meine Gedanken war definitiv beeinträchtigt, allerdings nur mäßig und gelegentlich.

2🞎 Ich hatte keinerlei Kontrolle über meine Gedanken.

**11 - Isolation von der Umwelt**

0🞎 Ich fühlte mich nicht von der Umwelt isoliert.

1🞎 Ich fühlte mich definitiv von der Umwelt isoliert, allerdings nur mäßig und gelegentlich.

2🞎 Ich fühlte mich völlig von der Umwelt isoliert.

**12 – Automatische Gehorsamkeit (im Sinne von Befolgen von Aufforderungen des Untersuchers)**

0🞎 Ich hatte eigene Willenskraft.

1🞎 Ich fühlte mich definitiv in eigener Willenskraft eingeschränkt, allerdings nur mäßig und gelegentlich.

2🞎 Ich fühlte mich völlig in eigener Willenskraft eingeschränkt.

**13 - Manierismen**

0🞎 Ich hatte nicht das Gefühl, ich muss seltsame, bizarre Bewegungen ausführen.

1🞎 Ich hatte definitiv das Gefühl, ich muss seltsame, bizarre Bewegungen ausführen, allerdings nur mäßig und gelegentlich.

2🞎 Ich hatte vollkommen das Gefühl, ich muss seltsame, bizarre Bewegungen ausführen.

**14 - Stereotypien**

0🞎 Ich hatte nicht das Gefühl, ich muss (sinnlose) Bewegungen wiederholen.

1🞎 Ich hatte definitiv das Gefühl, ich muss (sinnlose) Bewegungen wiederholen, allerdings nur mäßig und gelegentlich.

2🞎 Ich musste ständig (sinnlose) Bewegungen wiederholen.

**15- Dyskinesien**

0🞎 Ich hatte nicht das Gefühl, ich muss abnormale, unwillkürliche und schnelle Bewegungen ausführen, die ich nicht kontrollieren konnte.

1🞎 Ich hatte definitiv das Gefühl, ich muss abnormale, unwillkürliche und schnelle Bewegungen ausführen, die ich nicht kontrollieren konnte, allerdings nur mäßig und gelegentlich.

2🞎 Ich hatte vollkommen das Gefühl, ich muss abnormale, unwillkürliche und schnelle Bewegungen ausführen, die ich nicht kontrollieren konnte.

**16 - Posieren**

0🞎 Ich hatte nicht das Gefühl, ich muss plötzlich Posen einnehmen, die ich nicht kontrollieren konnte.

1🞎 Ich hatte definitiv das Gefühl, ich muss plötzlich Posen einnehmen, die ich nicht kontrollieren konnte, allerdings nur mäßig und gelegentlich.

2🞎 Ich hatte vollständig das Gefühl, ich muss plötzlich Posen einnehmen, die ich nicht kontrollieren konnte.

**17- Katalepsie**

0🞎 Ich hatte nicht das Gefühl, dass ich von anderen herbeigeführte Bewegungen nicht zurückändern kann.

1🞎 Ich hatte definitiv das Gefühl, dass ich von anderen herbeigeführte Bewegungen nicht zurückändern kann, allerdings nur mäßig und gelegentlich.

2🞎 Ich hatte ständig das Gefühl, dass ich von anderen herbeigeführte Bewegungen nicht zurückändern kann.

**18 - Hypotonus**

0🞎 Meine Muskeln waren nicht schlaff.

1🞎 Meine Muskeln waren definitiv schlaff, allerdings nur mäßig und gelegentlich.

2🞎 Meine Muskeln waren völlig schlaff.

**19 - Impulsivität**

0🞎 Ich habe keine plötzlichen und unpassenden emotionalen Reaktionen und Verhaltensweisen gezeigt, die ich später bereut habe.

1🞎 Ich habe definitiv plötzliche und unpassende emotionalen Reaktionen und Verhaltensweisen gezeigt, die ich später bereut habe, allerdings nur mäßig und gelegentlich.

2🞎 Ich habe ständig plötzliche und unpassende emotionalen Reaktionen und Verhaltensweisen gezeigt, die ich später bereut habe.

**20– Aggressivität gegen Menschen**

0🞎 Ich habe keine Menschen verbal oder körperlich angegriffen.

1🞎 Ich habe definitiv Menschen verbal oder körperlich angegriffen, allerdings nur mäßig und gelegentlich.

2🞎 Ich habe ständig Menschen verbal oder körperlich angegriffen.

**21 – Aggressivität gegen Gegenstände**

0🞎 Ich habe keine Gegenstände zerstört.

1🞎 Ich habe definitiv Gegenstände zerstört, allerdings nur mäßig und gelegentlich.

2🞎 Ich habe ständig Gegenstände zerstört.

**22 - Flacher Affekt**

0🞎 Ich konnte meine Gefühle gut zeigen.

1🞎 Mein Gefühlsausdruck war definitiv beeinträchtigt, allerdings nur mäßig und gelegentlich.

2🞎 Ich konnte meine Gefühle überhaupt nicht zeigen.

**23 - Verbigeration**

0🞎 Ich habe keine Wörter oder Sätze wiederholt, die inhaltlich nicht passten.

1🞎 Ich habe definitiv Wörter oder Sätze wiederholt, die inhaltlich nicht passten, allerdings nur mäßig und gelegentlich.

2🞎 Ich habe ständig Wörter oder Sätze wiederholt, die inhaltlich nicht passten.

**24 - autonome Dysregulation/vegetative Abnormalitäten**

0🞎 Ich habe nicht geschwitzt, gezittert, hatte erhöhte Temperatur, Blutdruck, Puls und/oder meine Atmung war flach.

1🞎 Ich habe definitiv geschwitzt, gezittert, hatte erhöhte Temperatur, Blutdruck, Puls und/oder meine Atmung war flach, allerdings nur mäßig und gelegentlich.

2🞎 Ich habe ständig geschwitzt, gezittert, hatte erhöhte Temperatur, Blutdruck, Puls und/oder meine Atmung war flach.

**25 - Valenz – Ich fand den Zustand schön**

0🞎 Ich fand den Zustand sehr schön.

1🞎 Ich fand den Zustand mäßig schön.

4🞎 Ich fand den Zustand schrecklich.

**Auswertungshinweise**

- Patienten mit Katatonie sollten in der Lage sein, eine informierte Zustimmung zu erteilen und diesen Fragebogen trotz ihrer psychopathologischen Symptome (z. B. Akinesie und/oder Amnesie) zu beantworten.
- Patienten mit Katatonie sollten angeben, wie sie ihre katatonen Symptome aktuell erleben oder in den letzten 7 Tagen erlebt haben.
- Die Patienten sollten nur dann mit der Auswertung fortfahren, wenn die Frage #0 mindestens mit 1: „teilweise“ beantwortet wurde.
- Der NSSC-Gesamtscore ergibt sich aus der Summe der Bewertungen für die Items #1-#24.
- Item #25 sollte nur für die Bewertung der Valenz herangezogen werden, um einen Einblick in die emotionale Valenz des Zustands zu erhalten.
